# Diagnostic and therapeutic approach in adult patients with traumatic brain injury receiving oral anticoagulant therapy: an Austrian interdisciplinary consensus statement

**DOI:** 10.1186/s13054-019-2352-6

**Published:** 2019-02-22

**Authors:** Marion Wiegele, Herbert Schöchl, Alexander Haushofer, Martin Ortler, Johannes Leitgeb, Oskar Kwasny, Ronny Beer, Cihan Ay, Eva Schaden

**Affiliations:** 1 Department of Anaesthesia, Critical Care and Pain Medicine, Division of General Anaesthesia and Intensive Care Medicine, Medical University of Vienna, Waehringer Guertel 18-20, 1090 Vienna, Austria; 2 Department of Anaesthesiology and Intensive Care Medicine, AUVA Trauma Centre Salzburg, Academic Teaching Hospital of the Paracelsus Medical University, Dr. Franz Rehrl Platz 5, 5020 Salzburg, Austria; 3 Ludwig Boltzmann Institute for Experimental and Clinical Traumatology, AUVA Research Centre, Vienna, Austria; 4 Central Laboratory, Klinikum Wels-Grieskirchen, Grieskirchner Str. 42, 4600 Wels, Austria; 5 Department of Neurosurgery, Krankenhaus Rudolfstiftung, Juchgasse 25, 1030 Vienna, Austria; 6 Department of Neurosurgery, Medical University of Innsbruck, Innrain 52, Christoph-Probst-Platz, 6020 Innsbruck, Austria; 7 University Departments of Orthopaedics and Trauma Surgery, Division of Trauma Surgery, Medical University of Vienna, Waehringer Guertel 18-20, 1090 Vienna, Austria; 8 Department for Surgery and Sports Traumatology, Kepler University Hospital–Med Campus III, Krankenhausstraße 9, 4020 Linz, Austria; 9 Neurocritical Care, Department of Neurology, Medical University of Innsbruck, Anichstrasse 35, 6020 Innsbruck, Austria; 10 Department of Medicine I, Clinical Division of Haematology and Haemostaseology, Medical University of Vienna, Waehringer Guertel 18-20, 1090 Vienna, Austria

**Keywords:** Anticoagulation reversal, Coagulation management, Idarucizumab, Intracranial hemorrhage, Non-vitamin K antagonist oral anticoagulant (NOAC), Platelet inhibitors, Prothrombin complex concentrate (PCC), Traumatic brain injury, Vitamin K antagonist (VKA)

## Abstract

There is a high degree of uncertainty regarding optimum care of patients with potential or known intake of oral anticoagulants and traumatic brain injury (TBI). Anticoagulation therapy aggravates the risk of intracerebral hemorrhage but, on the other hand, patients take anticoagulants because of an underlying prothrombotic risk, and this could be increased following trauma. Treatment decisions must be taken with due consideration of both these risks. An interdisciplinary group of Austrian experts was convened to develop recommendations for best clinical practice. The aim was to provide pragmatic, clear, and easy-to-follow clinical guidance for coagulation management in adult patients with TBI and potential or known intake of platelet inhibitors, vitamin K antagonists, or non-vitamin K antagonist oral anticoagulants. Diagnosis, coagulation testing, and reversal of anticoagulation were considered as key steps upon presentation. Post-trauma management (prophylaxis for thromboembolism and resumption of long-term anticoagulation therapy) was also explored. The lack of robust evidence on which to base treatment recommendations highlights the need for randomized controlled trials in this setting.

## Preamble

The intention of the following consensus statement is to provide pragmatic, clear and easy-to-follow clinical guidance for the diagnosis and treatment of adult patients with traumatic brain injury (TBI) and potential or known intake of oral anticoagulants. We aimed to cover all clinical questions from the patient’s admission to the outpatient clinic or emergency room until discharge.

Few randomized controlled trials have been performed in this setting. Therefore, the evidence base for making recommendations is limited. Nevertheless, there is an urgent need for guidance in clinical practice. When in doubt, clinicians tend to favor more diagnosis and more therapy. This has major implications, firstly regarding healthcare costs, and secondly regarding patient safety. Patients take oral anticoagulants because of prothrombotic risk, and the administration of procoagulant therapy may increase this underlying risk.

It is the responsibility of the treating physician to perform a thorough risk-benefit analysis for each individual patient before making clinical decisions.

## Background

European epidemiological data show that traumatic brain injury (TBI) mainly affects elderly patients [1, 2]. A considerable proportion of elderly individuals are on oral anticoagulants due to cardiovascular problems. TBI is associated with high rates of morbidity and mortality in older patients [3, 4]. The intake of oral anticoagulants aggravates the risk of intracerebral hemorrhage following trauma and of secondary progression of bleeding lesions [5]. The available literature suggests that bleeding rates differ between types of oral anticoagulants, but the data are not conclusive. Treatment with non-vitamin K antagonist oral anticoagulants (NOACs) has been reported to lower the risks of morbidity and mortality compared to vitamin K antagonists (VKAs) [6, 7]. On the other hand, aspirin (ASA) has been associated with the highest rates of intracerebral hemorrhage (ICH) upon admission [8].

An interdisciplinary group of Austrian experts was convened to answer clinical questions regarding the management of TBI patients with potential or known intake of oral anticoagulants, and to develop recommendations for best clinical practice.

## Methods

The task force for perioperative coagulation of the Austrian Society of Anaesthesiology, Resuscitation and Intensive Care Medicine (OEGARI) assembled a national expert committee comprising representatives of the OEGARI, the Austrian Society for Hematology and Medical Oncology (OeGHO), the Austrian Society for Laboratory Medicine and Clinical Chemistry (ÖGLMKC), the Austrian Society of Neurology (ÖGN), the Austrian Society for Neurosurgery (ÖGNC) and the Austrian Society for Traumatology (ÖGU).

The scope of this consensus statement is adult patients who experience isolated TBI while receiving anticoagulants. The term “traumatic brain injury (TBI)” is defined according to the underlying pathomechanism of injury irrespective of the severity of the trauma (TBI can be mild, moderate, or severe). If the initial CCT scan indicates intracranial bleeding, we use the term “intracranial hemorrhage (ICH)”; in this setting, such terminology is equivalent to “hemorrhagic TBI.” Notably, *spontaneous* ICH is beyond the scope of this document.

The term “anticoagulant” is not defined uniformly; our approach was to include platelet inhibitors (e.g., ASA, clopidogrel, prasugrel, ticagrelor), VKAs, and NOACs (dabigatran, apixaban, edoxaban, rivaroxaban). Other anticoagulants (low molecular weight heparins, unfractionated heparin, and other parenterally available anticoagulants) were excluded. We also elected not to include patients with congenital bleeding disorders. A PubMed literature research was performed for the period January 2007 to September 2018 using the following Medical Subject Heading (MeSH) terms: traumatic brain injury, brain injury, head injury, head trauma, craniocerebral injury, CCI, cerebral trauma, platelet, platelet function, Multiplate, PFA, platelet function analyzer, DOAC, NOAC, new oral anticoagulant, novel oral anticoagulant, antithrombotic therapy, anticoagulation, start, restart, commence, recommence, clinical trial, systematic review, and editorial.

To ensure clinical relevance, we developed recommendations in the form of answers to frequently asked questions. Due to the paucity of randomized controlled trials, the recommendations were mainly based on expert opinion and current clinical practice. Therefore, the use of the GRADE system was waived.

## Recommendations for best clinical practice

**Fig. 1:**
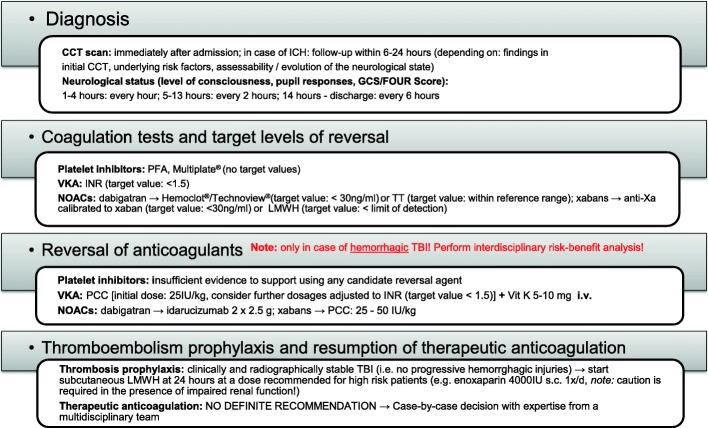
Best practice recommendations for the diagnosis and treatment of adult patients experiencing traumatic brain injury during treatment with oral anticoagulants

The recommendations are concisely summarized in Fig. 1.

### Diagnosis: Cranial computed tomography (CCT) scan and clinical findings

#### Clinical question: Should a CCT scan be performed in all patients with suspected or known TBI and potential or known intake of oral anticoagulants?

All patients with suspected or known TBI and potential or known intake of oral anticoagulants require a CCT scan irrespective of anamnesis or neurological examinations [e.g., Glasgow coma scale (GCS) score, FOUR score].

For TBI patients whose coagulation status is disrupted by anticoagulant therapy or other factors (e.g., liver dysfunction, hemophilia, thrombocytopenia), indications for CCT scan and observation are not clearly defined in the literature. Serious intracranial pathologies may occur in patients with minor head trauma but no additional risk factors. Several studies have shown an increased risk for intracranial pathologies in TBI patients with coagulation disorders, although these patients may exhibit normal neurological examination results and normal anamnesis (i.e., no apparent loss of consciousness, amnesia, or disorientation) [9–11]. As a result, recommendations for TBI patients with a coagulation disorder as the only risk factor for an intracranial pathology range from no routine initial CCT and discharge to CCT plus observation in the hospital [12–14].

Several international guidelines describe risk factors and categorize patients into low-, medium-, and high-risk categories for clinically significant intracranial pathologies. These risk factors refer mainly to the mechanism of injury, patient age, comorbidities, and the neurological exam. Internationally renowned guidelines [e.g., Canadian CT head rule, New Orleans Criteria, NICE clinical guideline, NEXUS II] report sensitivities close to 100% for the detection of intracranial pathologies requiring surgical intervention [15–17].

The heterogeneity of TBI patients taking anticoagulants complicates research and can make study results controversial. Of the available anticoagulants, only VKAs are proven to increase TBI mortality, and in several guidelines, these drugs are identified as an indication to perform a CCT [18–20]. For other anticoagulants (antiplatelet drugs, NOACs), some studies have reported increased risks of intracranial pathologies and mortality [7, 21–24]. In the absence of proven links, clear statements regarding the relative risks in patients taking antiplatelet drugs or NOACs cannot be made. However, we recommend performing a CCT in all patients with TBI and potential or known intake of oral anticoagulants.

#### Clinical question: Should a follow-up CCT scan be performed routinely? If yes, when?

Patients with TBI and potential or known intake of oral anticoagulants require a routine follow-up CCT scan only in case of ICH in the initial CCT. When indicated, the follow-up CCT should be performed between 6 and 24 h after the trauma.

The course of ICH in the presence of coagulation disorders cannot be predicted based on the literature. There is no clear evidence regarding repeated CCT scans in case of pathology on initial CCT in patients on antithrombotic therapy, resulting in a lack of widely accepted standards for the timing and number of repeat CCT scans. However, the decision when to schedule the follow-up CCT may be influenced by the findings of the initial CCT, underlying risk factors and the evolution of neurologic state.

#### Clinical question: Should a patient with a normal CCT scan be admitted for monitoring of the neurological state? If yes, for how long and what kind of monitoring should be used?

All patients with TBI and potential or known intake of oral anticoagulants with a normal CCT should be admitted and observed for at least 24 h after trauma. A follow-up CCT is indicated only in case of neurological deterioration (changes in GCS and pupil responses or FOUR score, as determined by specialists in neurology, trauma surgery, neurosurgery, or intensive care medicine). Neurological examinations should be performed every hour during the first 4 h, every 2 h during the following 8 h and every 6 h during the next 12 h.

Patients whose antithrombotic therapy comprises ASA monotherapy only may be discharged immediately under the following conditions: normal initial CCT scan, GCS 15, absence of other risk factors, and guaranteed observation by nursing home staff or suitably instructed close family/friends.

Due to numerous cases of delayed ICH in anticoagulated TBI patients with normal initial neurological examination and CCT scan findings, we recommend admission and observation for at least 24 h [25–29]. Patients who have ASA treatment as their only risk factor and meet the above conditions do not require admission after a normal CCT, due to a lack of studies showing increased risk for delayed ICH or mortality in these patients [30–32].

ICH can occur days or weeks after trauma, meaning that no protocol will be fully effective. Delayed bleeding has been reported to affect 0.2–6% of TBI patients on VKAs or clopidogrel with normal findings upon repeat CCT [25–28]. Observation for 24 h after trauma appears sufficient to detect the majority of clinically significant bleeding episodes [25–29].

For TBI patients on anticoagulants whose initial CCT findings are normal, routine performance of a repeat CCT appears to be of little benefit, hence the recommendation to scan only patients with neurological deterioration [33, 34]. GCS and pupil responses or the FOUR Score are suitable for neurological evaluation [35].

#### Clinical question: How should I proceed with patients with a normal CCT scan who cannot be examined neurologically?

All patients with TBI and potential or known intake of oral anticoagulants with a normal CCT scan who cannot be examined neurologically (e.g., due to intubation, sedation, or dementia) require a follow-up CCT within 6–24 h after trauma.

If the patient cannot undergo sufficient neurological examination (e.g., because they are intubated, sedated or demented), we recommend a repeat CCT within 6–24 h after trauma. The decision on exact timing may be influenced by underlying risk factors for delayed ICH or findings of the initial CCT scan.

## Coagulation tests and target levels of reversal

### Platelet inhibitors

#### Clinical question: Are platelet function tests [Platelet Function Analyzer (PFA®), impedance aggregometry (Multiplate®), VerifyNow®] capable of detecting and/or ruling out the presence of a platelet inhibitor?

Platelet function tests are capable of detecting and/or ruling out the presence of a platelet inhibitor. The intensity of platelet inhibition can be assessed, allowing an estimation of the bleeding risk. This might be useful especially when the patient’s medication is unknown.

Platelet function tests are established methods for detecting disorders of the primary hemostatic capacity (e.g., von Willebrand syndrome) and monitoring antiplatelet drugs. “Therapeutic ranges” have been established for the principal drugs [PFA®, seconds (Siemens Package insert 2012-10); Multiplate®, area under the curve (Roche Diagnostics Package insert 2016-12, V3.0 German); VerifyNow®, reaction units (Package insert)]. These enable clear statements as to whether the effect of treatment is within the therapeutic range. However, there is no evidence regarding residual inhibitory effects and the probability of accelerated bleeding when test results are outside the therapeutic range. Notably, these tests may help differentiate non-responders from non-compliant patients [36, 37].

As platelet count < 100G/l and/or hematocrit < 29% affect the results of platelet function tests (Siemens Innovance, package insert B4170G22C38 Rev.04–DE, 2012-05), they should not be used in these circumstances.

### Vitamin K antagonists (VKAs)

#### Clinical question: What is the target international normalized ratio (INR) in patients receiving VKAs when the initial CCT scan gives a positive result?

We recommend a target value of < 1.5 for INR.

The INR was developed to help ensure that VKAs are administered at doses within the therapeutic range [38]. The higher the INR value, the higher the bleeding risk. In patients with TBI and an INR > 2, the odds ratio for ICH has been reported as 2.59 compared to TBI patients not receiving warfarin [39]. In an observational study, Mason et al. measured the INR upon emergency room admission in 2934 TBI patients. The median INR of patients with an adverse outcome was only slightly higher than in those with a good outcome (2.5 vs 2.4). Univariate Poisson regression showed a significant positive association between INR and risk of adverse outcome (*p* = 0.029), but a significant association was not observed in patients with a GCS of 15 [40]. Data in the neurosciences literature suggest that INR > 1.2 is associated with worse outcomes in ICH [41].

Neurosurgical textbooks have recommended an INR < 1.4 for patients undergoing invasive procedures [42]. However, there are no robust data supporting this number. A moderate elevation of the INR is associated with only a modest deficiency in clotting factors that may be clinically insignificant. Three recent studies investigating the effect of prothrombin complex concentrate (PCC) on INR reversal used INR targets of 1.2–1.3 [43–45]. Powers et al. reported that, in patients receiving VKA treatment, stroke thrombolysis is possible at INR ≤ 1.7 [46]. This is in line with Hankey et al., who suggested a contraindication for stroke thrombolysis in VKA-treated patients with an INR > 1.7 [47]. However, Malloy et al. recommended INR < 1.5 for percutaneous image-guided interventions in patients on VKAs [48].

### Non-vitamin K antagonist oral anticoagulants (NOACs)

#### Clinical question: Should I use standard coagulation assays [PT, activated partial thromboplastin time (aPTT)] to detect and/or rule out NOAC intake?

Standard coagulation assays (PT, aPTT) are not suitable for detecting or ruling out the presence of a NOAC, except in specialized coagulation laboratories.

Standard coagulation tests (PT, aPTT) are not suitable for detecting a NOAC effect but, depending on the NOAC, they may indicate the presence of an anticoagulant. The effects of anticoagulant therapy on these tests vary according to the dose and the time elapsing since ingestion. However, meaningful deduction of the diagnostic possibilities based on standard coagulation tests can only be performed at specialized coagulation laboratories with expertise regarding the effects of NOACs, where specific reagents may be used [e.g., PT Neoplastin plus (Siemens, Marburg, Germany) makes the PT sensitive to rivaroxaban] [49–51].

#### Clinical question: Which test should be used to rule out the presence of dabigatran anticoagulation?

We recommend measuring thrombin time (TT) or dilute TT (dTT) to rule out the presence of dabigatran anticoagulation.

(a) A TT within the reference range excludes (remaining) dabigatran-associated anticoagulation.

(b) A dTT (Hemoclot®) level < 30 ng/ml excludes (remaining) dabigatran-associated anticoagulation.

A TT measurement within the normal range excludes the presence of dabigatran anticoagulation, since even low dabigatran concentrations (30–40 ng/ml) cause significant prolongation of the TT [47, 50, 52]. Due to the high sensitivity of the reagents, it is not possible to use TT measurements for quantitative determination of dabigatran or assessment of the risk of bleeding from dabigatran-mediated thrombin inhibition.

The diluted thrombin time (dTT) [e.g., Hemoclot® (Hyphen-BioMed, CoaChrom, Vienna, Austria), Technoview® (Technoclone, Vienna, Austria)] can be calibrated using dabigatran standards, and thus used for quantitative determination of dabigatran [53]. A dabigatran level < 30 ng/ml, likely to be observed > 4 h post-dose, excludes a relevant risk of bleeding. It should be noted that the detection limit of the available dTT assays is 30 ng/ml [54].

For release to stroke lysis, dabigatran levels < 62 ng/ml are cited as “safe for treatment thresholds” [55]. Further threshold dabigatran levels have been identified by the International Society on Thrombosis and Hemostasis (ISTH) as follows: < 30 ng/ml, acceptable for patients undergoing surgery with a high risk of bleeding; > 30 ng/ml, use antidote before surgery with a high risk of bleeding; > 50 ng/ml, use antidote when a patient is bleeding heavily [54].

#### Clinical question: Which test should be used to rule out the presence of apixaban, edoxaban, or rivaroxaban anticoagulation?

We recommend measuring anti-activated factor X (anti-Xa) activity—calibrated to low molecular weight heparin (LMWH) or the specific “xaban” of interest—to rule out apixaban, edoxaban, or rivaroxaban anticoagulation.

(a) Calibrated to LMWH, an anti-Xa activity below the detection limit of the respective laboratory excludes (remaining) xaban-associated anticoagulation.

(b) Calibrated to the particular xaban, anti-Xa activity < 30 ng/ml excludes (remaining) xaban-associated anticoagulation.

To exclude the effects of apixaban, edoxaban, and rivaroxaban (xabans), an anti-Xa test calibrated to LMWH can be used if the assay has been tested by specific standards for its sensitivity (lower detection limit) [56, 57].

Xaban levels can be specifically determined using an anti-Xa test calibrated for the particular xaban of interest [49–52, 58]. However, these specific anti-Xa tests are usually only offered by specialist coagulation laboratories and may not be available at all times of the day or week.

Hankey et al. concluded that a clinically relevant level of rivaroxaban or apixaban can be excluded if the anti-Xa level is below the limit of detection—typically > 5 h after the last dose [47, 54]. It should be noted that there are pharmacokinetic differences between xabans (e.g., peak and trough plasma levels). A xaban level < 30 ng/ml excludes a relevant risk of bleeding, but the detection limit of the available assays is 30 ng/ml [54].

“Safe for treatment thresholds” for stroke lysis are < 91 ng/ml for rivaroxaban and < 40 ng/ml for apixaban [55]. Further xaban thresholds from the ISTH are as follows: < 30 ng/ml, acceptable for patients undergoing surgery with a high risk of bleeding; > 30 ng/ml, administer PCC before surgery with a high risk of bleeding; > 50 ng/ml, administer when a patient is bleeding heavily [54].

### Reversal of anticoagulants

TBI without bleeding does not require pharmacotherapy for anticoagulation reversal. In case of pathological findings in the initial CCT scan, (temporary) cessation of anticoagulant medication and reversal of anticoagulation should be considered. This is because hemorrhagic lesions often progress during the early hours after trauma, and hemorrhagic progression of a contusion impairs clinical outcomes [59]. In this context, we favor the term “hemorrhagic TBI” [59].

#### Platelet inhibitors

Patients on clopidogrel are more likely to have progression of an initial ICH and a higher rate of neurosurgical intervention, in comparison with those receiving ASA [60]. However, the impact of platelet inhibitors on mortality remains unclear [61].

#### Clinical question: Should desmopressin (DDAVP) be administered to reverse the effect of platelet inhibitors?

There is no consistent evidence that DDAVP administration in hemorrhagic TBI patients on platelet inhibitors reduces progression of intracranial hematoma or improves neurologic outcome. Therefore, we cannot provide a clear recommendation for or against the use of DDAVP.

DDAVP prompts the release of von Willebrand factor and factor VIII from endothelial cells, accelerating platelet adhesion and improving primary hemostasis. Moreover, DDAVP stimulates the expression of glycoprotein receptor on the surface of platelets [62].

The effectiveness of DDAVP in reducing the progression of traumatic ICH is unknown. Only a few small studies have investigated the use of DDAVP in TBI or spontaneous ICH, and the results are unclear [63]. Naidech et al. reported a study in patients with spontaneous intracerebral hematoma and reduced platelet activity. In patients (*n* = 7) treated with DDAVP within 12 h of ICH symptom onset, a modest reduction in intracranial hematoma volume was observed (median 0.5 ml) [64]. In a prospective study, DDAVP was administered to 10 patients with ICH who had been receiving ASA. Although platelet function was improved, this effect was short-lived and not statically significant [65]. Kim et al. reported a retrospective study in TBI patients with intracerebral bleeding. Of 408 patients, 54 were on ASA or clopidogrel prior to trauma. Platelet transfusion and co-administration of DDAVP was not associated with a decreased risk of hemorrhage progression or mortality [66].

Despite the paucity of supporting data, DDAVP has been recommended in several guidelines for intracerebral bleeding in patients receiving platelet inhibitors [5, 67] and in trauma patients with von Willebrand disease [68].

#### Clinical question: Should tranexamic acid (TXA) be administered to reverse the effect of platelet inhibitors?

There is no evidence from randomized controlled trials that TXA improves outcome in hemorrhagic TBI. No studies have investigated the role of TXA in patients on platelet inhibitors. Therefore, we cannot provide a clear recommendation for or against the use of TXA in these patients.

Fibrinolysis has been identified as a major contributor to poor outcomes following trauma [69]. Profibrinolytic activation seems to be an important component of hemorrhage progression in TBI [70, 71]. Following the results of the CRASH-2 study, TXA administration has been implemented as standard therapy in many trauma centers worldwide [72–75]. In comparison with trauma, evidence to support using TXA in TBI patients is far less clear. Moreover, no studies have investigated TXA in TBI patients on platelet inhibitors.

In a subgroup analysis of TBI patients (*n* = 270) recruited in the CRASH-2 study, the effect of TXA on ICH in patients with TBI was analyzed. CCT scans performed before randomization and after 24–48 h showed comparable mean total hemorrhage growth with versus without TXA (TXA group 5.9 ± 26.8 ml; placebo group, 8.1 ± 29.2 ml) [76].

In a randomized, double-blind, placebo-controlled trial, Yutthakasemsunt et al. investigated 238 patients with moderate to severe TBI (GCS 4–12) and no coagulopathy. No significant difference in ICH progression was observed between the TXA group and placebo patients [RR = 0.65]. Risk of death from all causes and the risk of unfavorable outcome on the Glasgow outcome scale (GOS) were similar between groups (RR = 0.69 and RR = 0.76, respectively) [77].

Patients with traumatic ICH (*n* = 80; intracranial blood volume < 30 ml) were included in another randomized, placebo-controlled trial of TXA. Mean total hemorrhage expansion was significantly lower in the TXA group compared to placebo (1.7 ± 9.7 ml vs 4.3 ± 12.9 respectively; *p* < 0.001) [78].

Chakroun-Walha et al. performed a prospective, randomized trial of TXA in 180 TBI patients. Mortality and 28-day GOS were similar in patients who received or did not receive TXA. Thromboembolic events were five times more frequent in the TXA group but, importantly, there was a time delay of 8 h between trauma and TXA administration [79].

#### Clinical question: Should platelet concentrate be administered to reverse the effect of platelet inhibitors?

Platelet transfusion could conceivably reduce mortality following spontaneous intracerebral hemorrhage in patients receiving platelet inhibitors. However, no studies have demonstrated clear benefits in response to platelet transfusion in hemorrhagic TBI patients on platelet inhibitors. Therefore, routine use of platelet transfusion cannot be recommended.

A platelet count < 135 G/l in patients on antiplatelet therapy is predictive of both radiographic and clinical worsening [80]. Despite low platelet count being a predictor of poor outcome following TBI, platelet concentrate transfusion is controversial. This is because some data suggest that platelet concentrate transfusion in TBI patients on platelet inhibitors can be associated with poor outcomes [81, 82].

Downey et al. investigated the effect of platelet transfusion in a retrospective study of 328 TBI patients aged > 50 years on ASA or clopidogrel. Patients who received platelet transfusion had a similar mortality rate to those who were not treated with platelets (17.5% vs 16.7%, respectively; *p* = 0.85) [83]. In another retrospective study, Ducruet et al. analyzed 66 patients on antiplatelet therapy (ASA and/or clopidogrel) who suffered a primary ICH. Hematoma expansion was similar in transfused versus non-transfused patients [84]. Briggs et al. assessed the effect of platelet transfusion in TBI patients, 12 on ASA and 5 not on ASA. The ASA-induced component of platelet dysfunction but not the trauma-induced component was ameliorated by platelet transfusion [85].

In an open-label trial, patients with spontaneous supratentorial ICH who were receiving antiplatelet therapy were randomly assigned to platelet transfusion (*n* = 97) or standard care (*n* = 93). Patients receiving platelet transfusion had a higher risk of death or dependence at 3 months than the standard care group (OR 2.05; *p* = 0.0114). The percentage of patients with serious adverse events was higher with platelet transfusion versus standard care (42% vs 29%, respectively) [81]. Holzmacher et al. investigated the effect of platelet transfusion in TBI patients on ASA, clopidogrel, or dual platelet inhibition. Platelet transfusion significantly improved platelet dysfunction associated with ASA but not clopidogrel. A subgroup analysis of patients with an Injury Severity Score (ISS) > 15 revealed that platelet transfusion was associated with higher need for neurosurgical interventions, longer intensive care unit stay, and longer hospital length of stay. Moreover, platelet transfusion did not improve the Marshall CT score or mortality [82].

These findings are in line with a meta-analysis that included four studies of platelet transfusion in patients with traumatic ICH [18]. No clear survival benefit was observed with platelet transfusion.

### Vitamin K antagonists (VKAs)

#### Clinical question: Should VKAs always be reversed in case of hemorrhagic TBI?

Reversal of VKA anticoagulation is always recommended in patients with hemorrhagic TBI.

#### Clinical question: Should vitamin K be administered to reverse the effect of VKAs?

Vitamin K alone is not recommended as a reversal agent in patients with hemorrhagic TBI. However, it is recommended as an adjunct treatment in these patients. We suggest a dosage of 5–10 mg administered intravenously.

The major shortcoming of vitamin K for reversing the anticoagulant effects of VKAs is that reduction of INR to values less than 1.4 may take up to 24 h [86]. Nevertheless, in patients with non-life-threatening bleeding, intravenous vitamin K as monotherapy may be sufficient to achieve adequate hemostasis within 5 h [87].

In TBI patients with ICH who are receiving VKA therapy, vitamin K is essential for sustaining immediate reversal that is achieved using PCC [88]. To ensure a rapid response, we recommend intravenous rather than oral administration, and we suggest a dose of 5–10 mg [88].

#### Clinical question: Should PCC and/or plasma be used for reversal of VKAs?

Four-factor PCC is strongly recommended in preference to plasma for treating hemorrhagic TBI patients on VKAs. We recommend an initial dose of at least 25 IU/kg bodyweight. Further doses should be administered if needed to achieve INR < 1.5.

Plasma transfusion for reversal of VKA anticoagulation requires the administration of large volumes and is associated with risks of circulatory overload, acute lung injury, and immunosuppression. Numerous studies have shown that PCCs normalize INR faster than plasma, and there is evidence that quicker INR reversal reduces hematoma expansion [45, 89]. Thus, current guidelines recommend PCC in preference to plasma [67, 90].

PCCs are available with three or four vitamin K-dependent coagulation factors. Four-factor PCC appears more effective for normalizing INR induced in patients treated with VKAs [91–93]. The optimal dose of PCC for correction of INR depends on the INR. In one study, patients with TBI on VKA received either a low dose (25 IU/kg bodyweight) or a moderate dose (35 IU/kg) of PCC. The moderate dose was significantly more effective than the low dose in achieving INR < 1.5 (69% vs 12%; *p* < 0.001). Moreover, INR reversal was accomplished in 1.9 h in the moderate-dose group compared with 6.9 h in the low-dose group (*p* = 0.04) [94]. The dose of PCC required to normalize INR depends on the intensity of anticoagulation. Therefore, no specific dose can be recommended.

Majeed et al. reported a multicenter, retrospective study, of 140 patients with VKA-related intracerebral hemorrhage (INR > 1.5). Patients receiving plasma for VKA reversal (*n* = 40) showed greater progression of hematoma compared to those receiving PCC (mean hematoma volume, 64.5 vs 36.0 cm^3^; *p* = 0.021). The unadjusted OR for all-cause 30-day mortality in the PCC group was 0.40 (*p* = 0.021) compared to the plasma group. However, after adjusting for bleeding localization, age, and hematoma volume, the effect of PCC on mortality became non-significant [95]. In a randomized open-label trial, adults with VKA-associated ICH (INR ≥ 2.0) were treated with plasma (20 ml/kg bodyweight) or four-factor PCC (30 IU/kg). Significantly more patients in the PCC group than in the plasma group achieved INR ≤ 1.2 within 3 h of treatment [18/27 (67%) vs 2/23 (9%); *p* = 0.0003]. Hematoma expansion was reduced with PCC compared to plasma, although there was no significant between-group difference in mortality or GCS at 90 days [45].

Yanamadala et al. reported a study of patients undergoing emergency reversal of VKA anticoagulation using either plasma (*n* = 28) or PCC (*n* = 5). INR at presentation was similar between the two groups (plasma, 2.9; PCC, 3.1; *p* = 0.89). The time to reversal was significantly shorter in the PCC group (65 vs 256 min; *p* < 0.05) and, consequently, surgery was performed sooner in the PCC group [96]. Similarly, a retrospective study showed that PCC resulted in significantly faster INR reversal versus plasma (151.6 vs 485.0 min, respectively; *p* < 0.001). The incidence of ICH progression was decreased with PCC compared to plasma (17.2% vs 44.2%; *p* = 0.031) [97].

#### Clinical question: Should recombinant activated factor VII (rFVIIa) be used for the reversal of VKA anticoagulation?

The available evidence shows no benefit from using rFVIIa versus PCC for the reversal of VKA in hemorrhagic TBI.

Recombinant activated factor VII (rFVIIa) could, in theory, be an alternative option for rapid reversal of VKA anticoagulation in patients with ICH. Two small retrospective studies compared three-factor PCC and rFVIIa in this setting, and the results do not suggest that rFVIIa should be considered as preferable [98, 99]. In one of the studies, the time to INR reversal was similar with both treatments (PCC, 784 min; rFVIIa, 980 min), but INR rebound occurred more frequently in the rFVIIa group [99]. In the second study, INR reversal (≤ 1.3) within 1 h was achieved in 83% of patients treated with rFVIIa compared to 20% of those who received three-factor PCC. However, hematoma expansion occurred in a higher percentage of patients in the rVIIa group than in the PCC group (20% vs 11%) [98].

### Non-vitamin K antagonist oral anticoagulants (NOACs)

#### Clinical question: Should idarucizumab always be administered to a patient with hemorrhagic TBI and known intake of dabigatran?

The administration of idarucizumab depends on the coagulation tests available.

- If laboratory testing is not possible, administration of idarucizumab 2 × 2.5 g is recommended. Consider repeat dosing in patients with ongoing bleeding.
- A TT within the reference range excludes a (remaining) dabigatran-associated anticoagulant effect [see (a) in the section “Clinical question: Which test should be used to rule out the presence of dabigatran anticoagulation”]. In this case, the administration of idarucizumab is not required.
- A dTT (Hemoclot®) level < 30 ng/ml excludes a (remaining) dabigatran-associated anticoagulant effect [see (b) in the section “Clinical question: Which test should be used to rule out the presence of dabigatran anticoagulation”]. In this case, the administration of idarucizumab is not required.

#### Clinical question: Should PCC always be administered to a patient with hemorrhagic TBI and known intake of apixaban, edoxaban, or rivaroxaban?

The administration of four-factor PCC depends on the coagulation tests available.

- If laboratory testing is not possible, administration of four-factor PCC (25–50 IU/kg BW) is suggested unless more specific antagonists are available for routine clinical use (e.g., andexanet alfa). Consider repeat dosing in patients with ongoing bleeding.
- Calibrated to LMWH, an anti-Xa activity below the detection limit excludes (remaining) xaban-associated anticoagulation [see (a) in the section “Clinical question: Which test should be used to rule out the presence of apixaban, edoxaban, or rivaroxaban anticoagulation?”]. In this case, the administration of four-factor PCC is not required.
- Calibrated to the particular xaban of interest, an anti-Xa activity < 30 ng/ml excludes (remaining) xaban-associated anticoagulation [see (b) in the section “Clinical question: Which test should be used to rule out the presence of apixaban, edoxaban, or rivaroxaban anticoagulation?”]. In this case, the administration of four-factor PCC is not required.

Studies investigating NOAC reversal specifically in patients with hemorrhagic TBI are scarce. However, data are available from patients with ICH. Majeed et al. prospectively investigated 84 bleeding patients on rivaroxaban or apixaban who were treated with PCC for anticoagulation reversal. ICH was the most common site of bleeding (*n* = 59; 70.2%). PCC (median dose 2000 IU) was assessed as effective in 58 patients (69.1%), including 43 patients with ICH (72.9%) [100]. In a retrospective study of 27 NOAC-related bleeding events, 41% of which were ICH, a variety of different treatments were administered for anticoagulation reversal (PCC, activated PCC, plasma, and/or platelets), although no hemostatic therapy was administered in 29.6% of the episodes. Five thromboembolic events occurred, all of which were in patients who had received anticoagulation reversal treatment. There were six deaths, with a fatality rate of 45% among the patients with ICH [101]. Beynon et al. reported a retrospective study of ICH in 55 NOAC-treated patients, 33% of whom had TBI. NOAC reversal was not standardized, and specific antagonists were not available; 56% of the patients were treated with PCC. PCC therapy had no apparent effect on INR, and there was no difference in PCC administration between survivors and non-survivors (the overall mortality rate was 20%). It was concluded that the role of PCC as a reversal agent for NOAC-related ICH is unclear [102].

Several studies have reported that the rates of anticoagulation reversal are lower in bleeding patients on NOACs than in those on VKAs [7, 8, 103]. For example, in a multicenter study published by Kobayashi et al., the anticoagulant effect of NOACs in trauma patients with ICH was reversed in 13% of cases, compared with 47% for warfarin (*p* < 0.001) [8]. A similar difference in the pharmacological reversal rates for warfarin and NOACs was reported by Barletta et al. in a study of trauma patients [13.8% (NOAC) vs 48.1% (warfarin), *p* < 0.001] [103]. Prexl and colleagues performed a retrospective study of patients with TBI, and reversal agents were used in 24.2% of patients receiving NOACs compared with 84.4% of those receiving VKAs (*p* < 0.001) [7]. Despite these findings, clinical outcomes in patients receiving NOACs do not appear to be worse than those in patients receiving VKAs: Barletta et al. reported mortality rates of 4.3% and 5.9% in the two groups (*p* = 0.789), while Prexl and colleagues found a significantly lower mortality rate in patients taking NOACs compared to those on VKAs (3% vs 22%; *p* = 0.047) [7, 103].

The specific antibody idarucizumab is now available for the reversal of dabigatran-related anticoagulation. Pollack et al. reported a prospective study of this agent in 301 patients with serious bleeding while on dabigatran. ICH was present in 32.6% of these patients, and the median time to bleeding cessation was 2.5 h. At 90 days, thrombotic events had occurred in 6.3% of the patients, and there were no serious adverse safety signals [104].

For the reversal of xabans, andexanet alfa—a catalytically inactive recombinant form of factor Xa—has been developed. In May 2018, the US Food and Drug Administration (FDA) approved andexanet alfa for the reversal of apixaban and rivaroxaban. Approval from the European Medicines Agency (EMA) is still pending. However, there are no data regarding the use of andexanet alfa in patients with hemorrhagic TBI.

#### Clinical question: Should NOACs always be reversed in case of hemorrhagic TBI? If not, are there criteria to guide decision-making?

There are insufficient data in the literature to recommend NOAC reversal in all patients with TBI. There are also insufficient data to determine whether certain patients do not require anticoagulation reversal.

Studies of the risks and benefits of not reversing the NOAC effect in selected subpopulations with TBI are scarce. Case series have shown that elderly patients with TBI caused by a fall from low height (GCS ≥ 14) have favorable outcomes in the absence of a clear reversal strategy (no specific antidotes available) [7, 103, 105].

The decision to actively reverse the anticoagulant effects of NOAC in patients with TBI must be balanced against the risks associated with rapid anticoagulation reversal, the limited availability of specific reversal agents, and also the costs of treatment. The neurological and overall clinical condition of the patient should also be considered (e.g., imaging results, concomitant use of platelet inhibitors, hepatic and renal function, the potential for ongoing bleeding to cause central nervous system damage, and the potential need for surgery within the next 48 h).

Expert opinion based on clinical practice suggests that NOAC reversal is not required in the following circumstances: (1) negative initial CCT scan and a GCS ≥ 14, (2) open head injury where inspection of the injured scalp indicates normal coagulation status, and (3) unilateral chronic subdural hematomas and minimal or absent symptoms (GCS 15, slight headache, unilateral minimal weakness, reflex asymmetry, midline shift < 5 mm). A wait-and-observe strategy may be applied to patients meeting these criteria.

### Thromboembolism prophylaxis and resumption of therapeutic anticoagulation after hemorrhagic TBI

#### Clinical question: What is the optimal timing and preferred agent for pharmacological thromboembolism prophylaxis in patients after hemorrhagic TBI?

Considering the updated Brain Trauma Foundation guideline document and recently published literature, we recommend initiating thromboembolism prophylaxis 24 h after injury in patients who have a clinically and radiographically stable TBI. In addition, we recommend LMWH as the agent of choice, at a dose suitable for patients with a high risk of thrombosis (e.g., subcutaneous enoxaparin 4000 IU once daily).

Patients receiving anticoagulation therapy are at risk of progression of ICH following TBI of any severity [106]. On the other hand, these patients are also at significant risk of thromboembolic complications, both early and late after trauma. The reported incidence of venous thromboembolic events in isolated TBI varies from 3 to 25%, when thromboembolism prophylaxis is delayed or not administered [107, 108]. Notably, the risk of thromboembolic events increases with TBI severity [109]. It is difficult to quantify the risks of hemorrhagic progression and thromboembolic complications, but clinical decisions must be taken with due consideration of both these possibilities.

In trauma patients, the efficacy of pharmacological prophylaxis in preventing thromboembolic events is well established [110]. Regarding TBI, the updated Brain Trauma Foundation guideline recommends LMWH or unfractionated heparin (UFH) in combination with mechanical prophylaxis but the time frame for this treatment is not specified [111]. This issue was addressed in a recent systematic review and meta-regression analysis, which demonstrated no relationship between the rate of hemorrhagic expansion and timing of thromboembolism prophylaxis [112]. Fourteen studies showed that pharmacologic thromboembolism prophylaxis, administered 24–72 h after injury, is well tolerated in patients with stable TBI, and 4 studies suggested that administering thromboembolism prophylaxis within 24 h of injury does not lead to progressive traumatic ICH. The authors concluded that pharmacologic prophylaxis can be administered as early as 24–48 h following TBI without risk of increased hemorrhage, but some important limitations should be considered. Most of the studies selected patients with low hemorrhage risk according to the modified Berne-Norwood criteria [113]. Selection of patients with stable hemorrhagic lesions (i.e., no increase in size or number of lesions between admission and repeat neuroimaging 24 h later) was another method of ensuring low risk before initiating thromboembolism prophylaxis. Most importantly, TBI patients receiving oral anticoagulants have been excluded from studies determining the safety of post-traumatic thromboembolism prophylaxis [112]. Byrne et al. conducted a retrospective cohort study in patients with severe TBI [114]. Administration of thromboembolism prophylaxis within 72 h of trauma was associated with lower rates of both pulmonary embolism (OR, 0.48; 95% CI, 0.25–0.91) and deep vein thrombosis (OR 0.51; 95% CI, 0.36–0.72), but there was no increase in risk of late neurosurgical intervention or death when compared with late prophylaxis (i.e., after 72 h).

The prerequisite of a stable TBI as documented by repeat neuroimaging has been challenged recently. In a study by Frisoli et al., thromboembolism prophylaxis was begun either within 24 h of presentation or after 48 h [115]. The primary outcome of radiographic expansion occurred in 18% of patients in the early group compared to 17% in the delayed group (*p* = 0.83). Rates of thromboembolism (~ 2%) and mortality (~ 4%) were also similar in the two patient groups. The majority of patients had mild TBI, but outcomes were similar in patients with moderate and severe TBI.

In the absence of large-scale randomized trials showing whether LMWH or UFH is preferable for thromboembolism prophylaxis, the choice of agent is largely based on practitioner and institution preference. A large retrospective multicenter study in trauma patients demonstrated that LMWH was associated with a significantly lower rate of pulmonary embolism than UFH (1.4% vs 2.4%; OR, 0.56) [116]. In addition, LMWH has been associated with lower rates of heparin-induced thrombocytopenia and traumatic hematoma expansion [117, 118]. On the other hand, UFH has a shorter half-life and is more easily reversed. Therefore, UFH may be the preferred agent in high-risk situations with expanding hemorrhagic TBI lesions.

It is unclear whether existing protocols for thromboembolism prophylaxis after trauma are applicable to TBI patients receiving oral anticoagulants. There is a clear need for randomized controlled trials to determine the optimal timing, agent, and dose for pharmacologic thromboembolism prophylaxis in this setting, where the risks of both hemorrhagic progression and thromboembolic complications may be increased.

#### Clinical question: Should therapeutic anticoagulation be resumed after hemorrhagic TBI? If yes, what is the optimal timing?

There is insufficient evidence to support or discourage the resumption of therapeutic antithrombotic treatment following TBI. Expertise from a multidisciplinary team with experience of clinical practice should be sought to guide decision-making on a case-by-case basis.

After hemostasis is achieved and traumatic ICH has stopped, decisions on resuming anticoagulation therapy can be challenging because of the potential to increase the risk of hemorrhagic progression in the acute phase and the risk of bleeding in any future TBI. There is a paucity of evidence regarding the optimal timing for resuming oral anticoagulation after TBI [119]. This uncertainty is reflected by the results of a survey of practice patterns in patients with central nervous system hemorrhage and a history of atrial fibrillation and ischemic stroke [120]. The most common times for restarting anticoagulation after the index hemorrhage were 1 month (43.5%) followed by 1 week (33.7%), respectively. Only 13.3% of respondents indicated they would prefer an earlier restart time (3 days), and 8% indicated they would not restart anticoagulation. Interestingly, 47.7% of respondents indicated that they face dilemmas at least once per week concerning anticoagulation restart time and intensity, and 59.4% stated that they relied predominantly on intuition or past experience.

In a retrospective study, Albrecht et al. compared the risk of thrombotic and hemorrhagic events in patients who did or did not restart warfarin therapy during the 12-month period following hospitalization for TBI [121]. Resumption of warfarin treatment was associated with decreased risks of thrombotic events (RR, 0.77; 95% CI, 0.67–0.88) and hemorrhagic or ischemic stroke (RR, 0.83; 95% CI, 0.72–0.96). Although there was also an increased risk of hemorrhagic events (RR, 1.51; 95% CI, 1.29–1.78), the authors concluded that recommencing anticoagulation provided an overall net benefit for most patients. A more recent retrospective study showed that there is no additional risk of neurological deterioration related to the administration of anticoagulation within 60 days after injury [122]. In this study, intravenous heparin was the most commonly used agent (70.8%) for therapeutic anticoagulation.

Avoidance of therapeutic anticoagulation for at least 14 days post-TBI in patients without mechanical heart valves might decrease the risk of progression of traumatic hemorrhagic lesions. However, results from observational studies and retrospective analyses indicate that patients with a history of prior antithrombotic therapy experience thromboembolic complications significantly earlier after TBI, with a peak in the first 10 days post-trauma [123]. A recent literature review by Tykocki and Guzek provides evidence that resuming antithrombotic therapy early (range 3–17.5 days) following TBI may carry an acceptably low risk of hemorrhagic complications, and that the risk of complications may be lower with NOACs than with VKAs [124].

More robust data are available regarding the optimal time window for initiating anticoagulant treatment after *spontaneous* intracerebral hemorrhage in patients receiving anticoagulants. A retrospective study concluded that resumption should be delayed by at least 10 weeks to avoid the risk of early, recurrent hemorrhage [125]. In contrast, a systematic review of data from 63 publications suggested that anticoagulation in high-risk patients may be restarted 3 days from the time of the index hemorrhage [126]. A recent observational study investigated the resumption of antithrombotic treatment in 2619 patients with atrial fibrillation and intracerebral hemorrhage [127]. The benefits of anticoagulation therapy (reduced risk of vascular death and nonfatal stroke in high-risk patients) seemed to be greatest when it was resumed 7–8 weeks after intracerebral hemorrhage, and there was no significant increase in the risk of severe hemorrhage. A randomized controlled trial of anticoagulant use in atrial fibrillation patients who have had an intracerebral hemorrhage is currently in progress [128].

We advise careful consideration on a case-by-case basis, with a strong emphasis on specialist consultation. A multidisciplinary team should first consider the indication for anticoagulation. Patients with the greatest need for anticoagulation (e.g., those with mechanical heart valve prosthesis or antiphospholipid syndrome with recurrent thromboembolic events; Table 1) clearly require the resumption of anticoagulation. In selected cases, heparin-bridging therapy may be considered as an interim measure, but this should not be applied routinely given the possible risk of major bleeding [129, 130]. In atrial fibrillation, risk prediction tools including the CHA2DS2VASc and HASBLED score can help define the risk:benefit ratio of anticoagulation therapy [131]. However, these tools have not been validated for TBI patients with preinjury anticoagulation therapy. Furthermore, although NOACs are reported to carry a lower risk of spontaneous ICH than VKAs in atrial fibrillation patients [132], there are insufficient data to determine their usefulness as alternatives after hemorrhagic TBI. In agreement with international guidelines for the management of spontaneous intracerebral hemorrhage [87, 133], therapeutic anticoagulation may be continued after 10–14 days after TBI in patients with a stable injury and a high risk of cerebral ischemia (i.e., those with mechanical valve prosthesis or non-valvular atrial fibrillation and a CHA2DS2VASc score ≥ 4). In patients with moderate or low risk of thromboembolic events, it may be more appropriate to resume anticoagulation after 4–8 weeks.

**Table 1:** Indications for oral anticoagulation in patients at risk of venous thromboembolism (modified from Watzke et al. 2013) [134]

Low thromboembolic risk	High thromboembolic risk
Platelet inhibitors	Platelet inhibitors
▪ CHD or other cardiovascular diseases (cerebrovascular disease, PAD) without complications	▪ CHD or other cardiovascular diseases with complications or additional risk factors (ischemic cardiomyopathy, St.p. cardiac decompensation, diabetes mellitus, cerebrovascular disease, PAD, renal impairment)
▪ Diabetes mellitus with increased cardiovascular risk	▪ St.p. surgical or interventional procedures in patients with CHD, PAD, or cerebrovascular disease within the last year (e.g., coronary stent)
	▪ Acute coronary syndrome or myocardial infarction during the last year
VKAs and NOACs	VKAs and NOACs
▪ Non-valvular atrial fibrillation and CHADS2 score or CHADS2-VA2SC score ≤ 3 without stroke	▪ Non-valvular atrial fibrillation and CHADS2 score or CHADS2-VA2SC score > 3 or St.p. stroke
▪ Previous venous thromboembolism (> 3 months ago)	▪ Atrial fibrillation
▪ Mechanical aortic valve prosthesis without other risk factors (atrial fibrillation, cardiomyopathy, CHD, PAD, diabetes mellitus, age > 75 years, stroke)	▪ Mechanical mitral valve prosthesis or other mechanical valve prostheses with additional risk factors, particularly atrial fibrillation or St.p. stroke
	▪ Venous thromboembolism during the last 3 months

*CHD* coronary heart disease, *NOACs* non-vitamin K antagonist oral anticoagulants, *PAD* peripheral arterial disease, *VKAs* vitamin K antagonists

## Conclusions

The intention of this consensus statement was to provide pragmatic, clear, and easy-to-follow clinical guidance for the management of adult patients with TBI and potential or known intake of oral anticoagulants. We aimed to cover pertinent questions from the patient’s admission to the outpatient clinic or emergency room until discharge. The evidence base for making recommendations is limited by the scarcity of randomized, controlled trials in this setting. As a result, there has to be a strong emphasis on expert opinion and clinical experience. When in doubt, clinicians tend to favor more diagnosis and more therapy. This approach is likely to increase costs while potentially delaying the administration of treatment. On the other hand, due consideration of the potential risks and benefits is necessary to ensure optimal clinical outcomes. We hope that clinicians find the recommendations contained within this paper helpful when managing their patients.

## Abbreviations

anti-Xa: Anti-activated factor X
aPTT: Activated partial thromboplastin time
ASA: Aspirin
CCT: Cranial computed tomography
DDAVP: Desmopressin
dTT: Diluted thrombin time
EMA: European Medicines Agency
FDA: Food and Drug Administration
FOUR: Full Outline of UnResponsiveness
GCS: Glasgow coma scale
ICH: Intracranial hemorrhage
INR: International normalized ratio
ISTH: International Society on Thrombosis and Haemostasis
LMWH: Low molecular weight heparin
NOAC: Non-vitamin K antagonist oral anticoagulant
OEGARI: Austrian Society of Anaesthesiology, Resuscitation and Intensive Care Medicine
PCC: Prothrombin complex concentrate
PFA: Platelet function analyzer
PT: Prothrombin time
rFVIIa: Recombinant-activated factor VII
St.p.: Status post
TBI: Traumatic brain injury
TT: Thrombin time
TXA: Tranexamic acid
UFH: Unfractionated heparin
VKA: Vitamin K antagonist
